# Markerless Video-Based Gait Analysis for Motor Phenotyping in Individuals with Schizophrenia Using Interpretable Machine Learning

**DOI:** 10.3390/diagnostics16162585

**Published:** 2026-08-15

**Authors:** Posen Lee, Hao-Shan Wang, Shih-Yen Hsu, Chin-Hsuan Liu

**Affiliations:** 1 Department of Occupational Therapy, I-Shou University, Kaohsiung 84001, Taiwan; posenlee@isu.edu.tw; 2 Department of Radiology, Chi Mei Medical Center, Tainan 71004, Taiwan; wre880223@gmail.com; 3 Department of Information Engineering, I-Shou University, Kaohsiung 84001, Taiwan; 4 School of Occupational Therapy, College of Medicine, National Taiwan University, Taipei 100225, Taiwan

**Keywords:** schizophrenia, markerless video-based gait analysis, motor phenotyping, MediaPipe Pose, gait features

## Abstract

**Background/Objectives:** Motor abnormalities are frequently observed in schizophrenia, but accessible methods for objective gait quantification remain limited. This exploratory controlled-setting study examined whether markerless smartphone-based video analysis combined with interpretable machine learning could quantify gait-related motor phenotypes in individuals with schizophrenia. **Methods:** Gait videos were collected from 100 individuals with schizophrenia and 35 healthy controls using a single-site, single-device, standardized recording setup. MediaPipe Pose was used to extract skeletal landmarks and derive 12 image-plane spatiotemporal and estimated two-dimensional knee-kinematic gait features. After temporal segmentation and quality control, 404 usable gait segments derived from 135 participants were analyzed as repeated segment-level observations. Decision Tree and Support Vector Machine models were applied for exploratory segment-level group-separation analysis using segment-wise 15-fold cross-validation after the full post-quality-control dataset had been balanced before fold allocation. **Results:** Several extracted gait features differed between groups, particularly image-plane ankle displacement, mean step displacement, displacement velocity, step characteristics, and knee-joint motion. In the Decision Tree model, image-plane ankle displacement served as the primary root node, indicating its central role in internal segment-level group separation. However, the healthy control group was substantially younger and not age-matched. In addition, the segment-level statistical comparisons did not account for within-participant clustering. Accordingly, the reported *p* values and confidence intervals may overstate statistical precision. Separately, segment-wise cross-validation allowed segments from the same participant to occur across folds and resampling was performed before fold partitioning. Because oversampling was performed with replacement, duplicated segment instances could also occur across training and validation folds. Consequently, the statistical findings should be interpreted as exploratory segment-level patterns rather than participant-level inference, and the machine-learning performance estimates should be regarded only as potentially optimistic apparent internal segment-level results and should not be regarded as evidence of participant-level generalization, diagnostic validity, screening accuracy, or clinical applicability. **Conclusions:** Markerless video-based gait analysis with interpretable machine learning may provide a feasible research-support approach for quantifying gait-related motor phenotypes in individuals with schizophrenia. These findings should not be interpreted as evidence for participant-level clinical classification, diagnostic or screening validity, clinical utility, or readiness for deployment, or as proof of cross-device or cross-environment reproducibility. Future studies require matched controls, psychiatric comparison groups, subject-wise validation, external datasets, calibrated gait measures, systematic cross-configuration reproducibility testing, and privacy-preserving data governance.

## 1. Introduction

Schizophrenia is a severe psychiatric disorder with neurodevelopmental features, traditionally defined by disturbances in perception, thought, affect, and behavior [1]. In addition to these core clinical manifestations, motor abnormalities have increasingly been recognized as clinically relevant features of the disorder [2]. Such abnormalities may include spontaneous movement disturbances that have been reported across different stages of schizophrenia, including in some first-episode and antipsychotic-naïve samples [1,2,3]. Neurological soft signs provide one of the most established lines of evidence for these motor-related alterations and have been discussed as both trait-related and state-related indicators in schizophrenia research [2,4]. These findings have led some researchers to propose motor abnormalities as potential markers of neurodevelopmental alterations and illness vulnerability in schizophrenia [1,2,5].

Among these motor manifestations, impairments in interlimb coordination, postural regulation, and gait patterns have been reported in individuals with schizophrenia [6]. Gait is a complex, habitual full-body behavior that requires coordinated sensorimotor control, balance regulation, proprioceptive feedback, and bilateral limb coordination [7]. Subtle deviations in these processes may therefore provide information about broader sensorimotor and integrative dysfunction [8]. From an embodied cognition perspective, movement is not merely a peripheral motor output but may also reflect broader disturbances in self-processing and sensorimotor integration [9]. Recent studies linking movement markers with anomalous self-experience suggest that motor abnormalities and self-disturbances may represent related dimensions of integrative dysfunction [7,8,9]. These findings support the systematic investigation of quantifiable gait parameters as gait-related motor phenotypes in individuals with schizophrenia.

Despite their clinical relevance, motor abnormalities in individuals with schizophrenia remain underrepresented in routine psychiatric assessment and rehabilitation-oriented monitoring [3]. Current psychiatric assessments rarely include quantifiable full-body movement variables, and commonly used clinical tools provide limited information about dynamic gait and postural control [2]. Objective assessment of postural control and gait dynamics may provide useful information for clinical research, rehabilitation-oriented monitoring, and evaluation of intervention outcomes [10,11,12]. However, conventional movement assessment often requires specialized equipment, trained personnel, or manual annotation [11]. These requirements increase time and resource demands and may limit scalability and consistency in large-scale studies [10,13].

Recent advances in computer vision and image processing have made it possible to extract human movement information from videos recorded with standard cameras [13]. By reducing the need for wearable sensors or laboratory-based markers, markerless approaches may simplify data collection and reduce interference with natural walking behavior [14]. These methods can generate quantitative descriptors of movement patterns from video sequences, although their accuracy and generalizability depend on camera setup, algorithm selection, and validation procedures [10,13,14,15,16]. Pose-estimation models such as OpenPose [17], BlazePose [18], and YOLO-Pose [19] automatically detect body landmarks and track their positions across video frames. In a comparative study of markerless pose-estimation models for lower-limb kinematics, MediaPipe Pose showed favorable performance relative to OpenPose and MMPose in that specific application [20]. Although OpenPose has been widely used in movement analysis, its computational demands may limit deployment in resource-constrained or mobile settings [19]. Moreover, some markerless workflows still require manual processing to derive spatiotemporal outcomes, which can reduce workflow efficiency and limit broader use.

Building on previous work using MediaPipe Pose for lower-limb kinematic assessment, the present study applied this markerless pose-estimation framework to extract body landmarks from smartphone-based gait videos [21,22]. To address the need for accessible gait quantification, we developed an automated video-based gait analysis framework using a standard smartphone camera and MediaPipe Pose. The framework automatically extracts 12 image-plane spatiotemporal and estimated two-dimensional knee-kinematic gait features. These features were then integrated into interpretable machine-learning models to explore segment-level group separation between individuals with schizophrenia and healthy controls. A graphical user interface was also developed to support visualization, structured review, and export of extracted gait features for research use.

The methodological contribution of this study is the development and preliminary evaluation of an integrated, smartphone-based markerless gait-analysis framework for schizophrenia research. Unlike approaches that directly classify raw video frames using less interpretable deep-learning representations, the proposed framework converts MediaPipe Pose landmarks into 12 predefined and clinically interpretable image-plane gait descriptors. These descriptors are subsequently examined using interpretable machine-learning models and presented through a research-oriented graphical interface. The framework is intended to support exploratory gait-related motor phenotyping under controlled recording conditions and is not proposed as a clinical diagnostic or screening system.

The purpose of this study was to examine whether markerless video-based gait analysis combined with interpretable machine learning can provide objective and accessible quantification of gait-related motor phenotypes in individuals with schizophrenia. Because this was an exploratory controlled-setting study with a non-age-matched healthy comparison group, the aim was to characterize gait-feature differences between the enrolled groups rather than to establish schizophrenia-specific gait biomarkers. We hypothesized that image-plane spatiotemporal and estimated knee-kinematic descriptors derived from smartphone videos would reveal measurable group-level gait-feature patterns and provide a feasible research-support framework for future motor-phenotyping studies.

## 2. Materials and Methods

### 2.1. Research Ethics and Data Governance

This study was conducted in accordance with the Declaration of Helsinki and was approved by the Institutional Review Board of Kaohsiung Municipal Kai-Syuan Psychiatric Hospital, Kaohsiung, Taiwan (protocol code: KSPH-2022-14; approval date: 28 April 2023). After receiving comprehensive verbal and written explanations of the study procedures, all participants provided written informed consent before data collection began. Participants were informed that the video-based gait analysis was conducted solely for research purposes and for quantitative characterization of motor function under controlled recording conditions. The video-based system was not designed or approved for clinical diagnosis, screening, covert mental-health inference, employment assessment, insurance decision-making, school screening, legal decision-making, or surveillance use. No automated decision was returned to participants or clinicians for clinical use.

To protect participant privacy, all video data and extracted gait features were de-identified before analysis. Personal identifiers were removed from the analytical dataset and replaced with study codes. Original video files were stored in access-restricted and encrypted storage, and access was limited to authorized research personnel. Extracted pose landmarks and gait features were used for statistical and machine learning analyses without retaining directly identifiable personal information in the modeling dataset. Raw videos were not included in the modeling dataset and were not publicly shared because gait videos may contain potentially identifiable information.

### 2.2. Participants and Eligibility Criteria

This was an exploratory cross-sectional controlled-setting study using convenience sampling. Participants with schizophrenia were recruited from Kaohsiung Municipal Kai-Syuan Psychiatric Hospital, and healthy controls were recruited from I-Shou University between 2020 and 2024. The sample size was determined by the availability of eligible participants during the study period and was considered appropriate for preliminary methodological evaluation rather than confirmatory hypothesis testing.

Participants in the patient group had a documented diagnosis of schizophrenia according to DSM-5 criteria, established by a board-certified psychiatrist based on clinical interview and medical records. All patients had received a stable medication dose during the month prior to data collection. The clinical variables available for the schizophrenia group included age at illness onset, illness duration, and confirmation that the medication dose had remained stable during the preceding month. However, detailed antipsychotic class, chlorpromazine-equivalent dose, duration of antipsychotic exposure, standardized extrapyramidal symptom ratings, standardized psychiatric symptom-severity scores, standardized illness-stage indicators beyond the available age-of-onset and illness-duration data, physical activity measures, and detailed information regarding relevant comorbidities were not systematically collected. Because these variables were not available retrospectively, they could not be reliably reconstructed for the present analysis. Consequently, medication-adjusted, extrapyramidal-symptom-adjusted, symptom-severity-adjusted, physical-activity-adjusted, or comorbidity-adjusted analyses could not be conducted, and their potential effects could not be disentangled from the observed gait-feature differences. Before enrollment, participants were screened to confirm the absence of major surgery, musculoskeletal disorders, or lower-limb discomfort that could interfere with gait performance.

Exclusion criteria included the presence of arrhythmia during usual walking, increased risk of cardiopulmonary failure or respiratory distress during walking, and a history of myocardial infarction or unstable angina within the preceding month. The healthy control group included 35 undergraduate university students recruited for comparison. Eligibility for the healthy control group was based on self-reported absence of psychiatric disorder history; no structured psychiatric diagnostic interview was conducted for the control participants. Control participants were additionally screened to exclude neurological diseases, musculoskeletal disorders, vestibular problems, visual problems that could affect walking or pose estimation, recent injuries, and current use of medications that could influence gait performance. The same gait-related exclusion criteria were applied before gait assessment. The healthy control group was not age-matched to the schizophrenia group. This sampling strategy was used for exploratory comparison but limits causal interpretation of group differences because age-related gait variation could not be separated from schizophrenia-associated motor features.

### 2.3. Smartphone-Based Gait Analysis Pipeline

This study developed a smartphone-based gait analysis pipeline for exploratory group-level characterization of gait-related motor phenotypes in individuals with schizophrenia and healthy controls. The pipeline consisted of five steps: standardized smartphone video acquisition, temporal segmentation and quality control, MediaPipe Pose landmark extraction, computation of 12 image-plane gait descriptors, and exploratory segment-level machine-learning analysis. A separate PyQt5-based interface was developed to support visualization and export of the extracted features, as described in Section 2.10.

**Figure 1:**
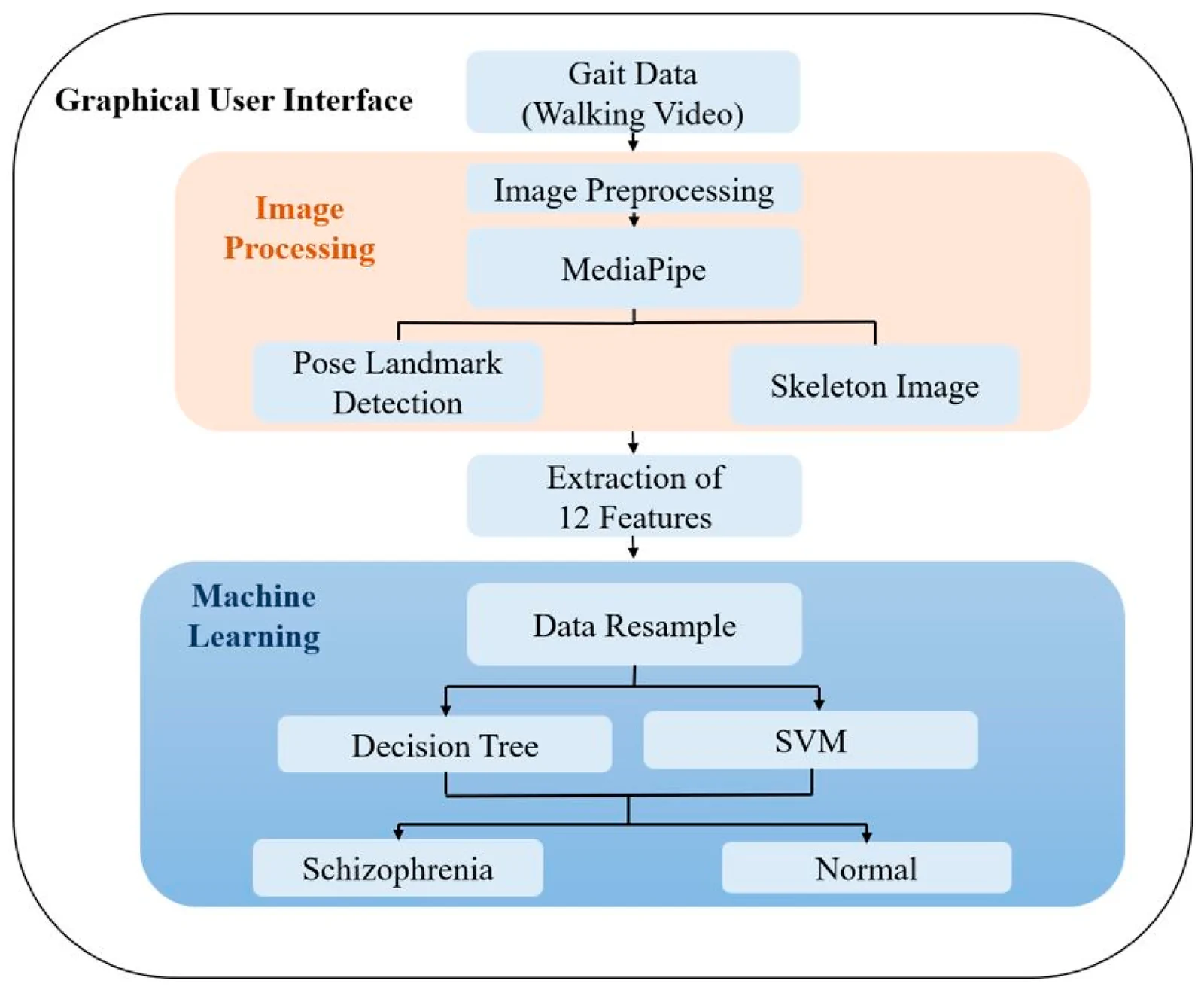
Flow of research.

In summary of the aforementioned methods, this study developed a PyQt5-based user interface that integrates computer vision and ML (Figure 1). The system utilizes MPP to extract pose landmarks from real-time or recorded videos, which a trained exploratory machine-learning model then summarizes as research-only segment-level output. This auxiliary tool automatically generates gait data to support exploratory characterization of group-level gait-feature differences.

### 2.4. Experimental Design

**Figure 2:**
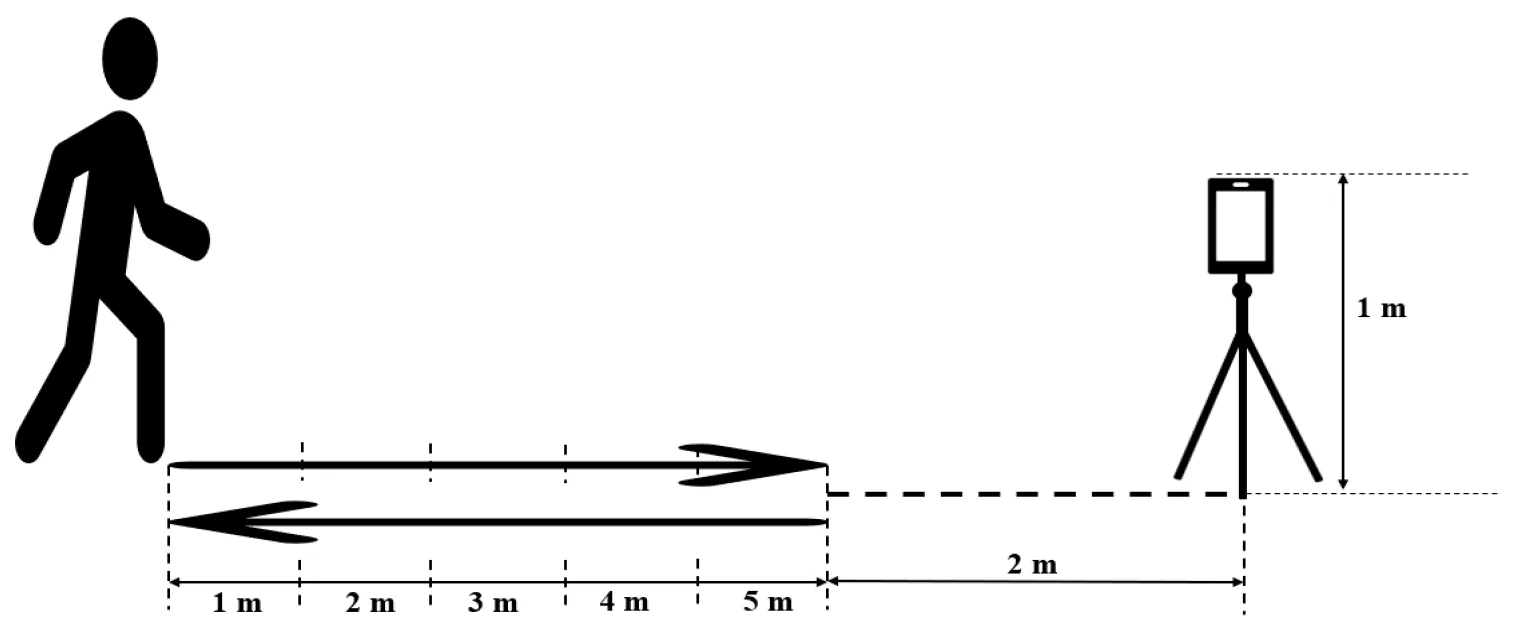
The experimental configuration.

The experimental configuration is illustrated in Figure 2. To standardize the recording environment, the walking path was 5 m in length, flat, level, and free of obstacles, with floor markings placed at 1 m intervals to guide participants along a consistent straight path. The walking path was operationally defined using two floor markers. The starting point was defined as the 0 m floor mark at the far end of the walking path, whereas the endpoint was defined as the 5 m floor mark closer to the camera. Gait videos were recorded using the rear camera of an iPhone 12 (Apple, Cupertino, CA, USA) at 1080p resolution and a fixed frame rate of 30 frames per second. The smartphone was mounted on a fixed tripod, and the camera was positioned 1 m above the ground and 2 m behind the 5 m endpoint of the walking path. The camera lens was oriented toward the centerline of the walking path so that participants walked directly toward the camera during the analyzed segments. All recordings were conducted indoors under stable ambient lighting conditions. Strong backlighting, direct sunlight, glare, and prominent shadows were avoided to support consistent body-landmark detection by MediaPipe Pose (version 0.10.21; Google LLC, Mountain View, CA, USA). Camera height, distance, orientation, and recording procedures were kept constant across participants within this single-site, single-device dataset. These controls were intended to reduce acquisition variability within the present study. Camera model, image resolution, frame rate, lens field of view, camera height, camera-to-participant distance, orientation, lighting, background, and recording site were not systematically varied. Therefore, the protocol did not evaluate robustness to camera-configuration changes or reproducibility across devices and recording environments.

Participants were instructed to walk at their usual comfortable speed without running or intentionally modifying their gait pattern. The same standardized verbal instruction was used for all participants. Short rests were allowed between trials when needed to minimize fatigue. No assistive devices were used during the recorded trials. Body height and body weight were not used to calibrate the image-plane gait descriptors; therefore, the extracted displacement-related variables were interpreted as relative pixel-based measures rather than body-size-normalized biomechanical parameters. No physical reference length, floor-plane transformation, camera intrinsic or extrinsic calibration, or conversion from pixels to metric units was applied. Consequently, identical physical movement recorded under a different resolution, framing, camera distance, camera height, or lens field of view could produce different numerical values for the pixel-based spatial descriptors.

All participants were instructed to wear walking shoes rather than slippers during data collection. Before formal video recording, each participant completed one practice walking trial along the 5 m path to become familiar with the walking path, task instructions, and turning procedure. The practice trial was not included in the final analysis. Each participant then completed three formal repetitions of walking toward and away from the camera. Because one video was recorded for each 5 m traversal, up to six raw videos could be obtained from each participant. For the present analysis, only toward-camera traversals were considered candidate gait segments because this direction provided a more consistent image-plane perspective for feature extraction. Return-walking segments, turning phases, preparatory movements, and post-walking pauses were excluded. No manual spatial cropping of the video frame was performed before pose estimation; the original full-body video frames were used for landmark detection.

### 2.5. MediaPipe Pose Detection

**Figure 3:**
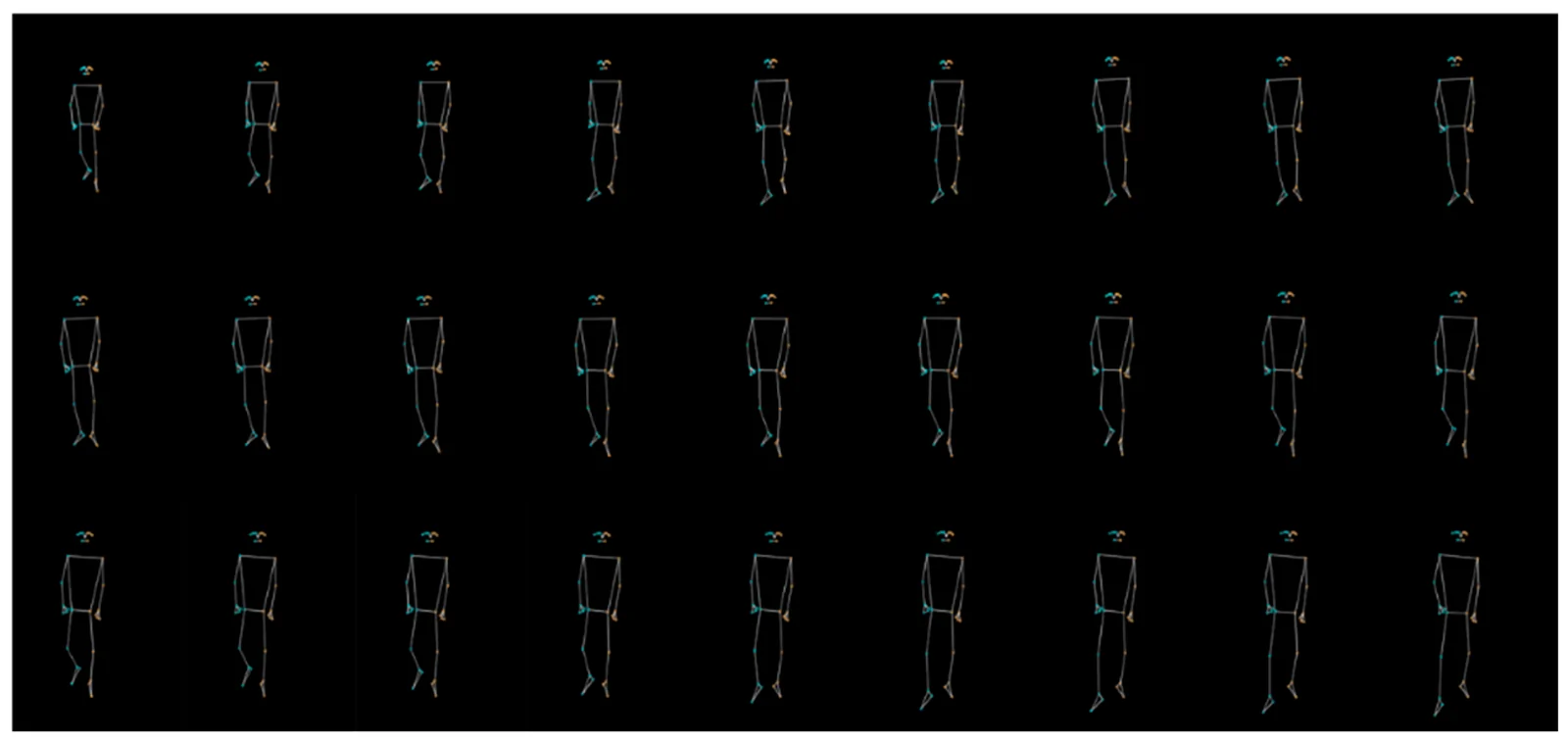
The holistic skeleton diagrams.

MPP is an open-source cross-platform framework provided by Google that utilizes ML methods to build various detection pipelines (e.g., hand, face landmarks, and pose detection) in video format. In this study, MPP utilizes the BlazePose model to extract 33 landmarks from the human body, as illustrated in Figure 3. BlazePose is a lightweight ML architecture designed to achieve real-time performance on mobile phones and PCs using CPU inference.

The construction of holistic skeleton diagrams with the background removed is shown in Figure 3. Subsequently, the estimated MPP landmarks were utilized to calculate 12 distinct gait features, as detailed in Table 1.

### 2.6. Extraction of Image-Plane Gait Features

The estimated MPP landmarks were utilized to calculate 12 distinct gait features (Table 1). The 12 gait features were computed from two-dimensional image-plane landmark coordinates derived from MPP. Because no camera calibration, depth reconstruction, or conversion to metric units was performed, displacement-related variables were interpreted as relative image-plane descriptors rather than calibrated biomechanical measures. The pixel-based spatial variables were not normalized to image dimensions, body size, or a physical reference length. Accordingly, their numerical scales were specific to the fixed resolution, framing, camera geometry, and participant-to-camera relationship used in the present study. The coordinate origin was located at the upper-left corner of the image frame, with the *x*-axis increasing to the right and the *y*-axis increasing downward. To optimize the quantification of gait features and improve within-dataset perspective uniformity, only video segments of participants walking toward the camera were analyzed. This restriction reduced variation in viewing direction but did not eliminate perspective distortion or establish measurement invariance across camera configurations. The calculation method for each feature is described as follows:

(1)  Step Count:

This feature identifies the relative position of the feet by comparing the vertical coordinates of the left (L) and right (R) ankles. Since the MPP origin is located at the top-left corner, a lower coordinate value indicates a higher vertical position, representing the lifted ankle. The step count specifically counts the number of times the magnitude relationship between the left and right ankle coordinates changes, reflecting the number of alternating steps taken.

To quantify the transitions, an indicator variable Si is defined for each frame i, where Li and Ri denote the vertical coordinates of the left and right ankles, respectively.

(1) *S_i_* = {1, *if L_i_* < *R_i_* 0, *if L_i_* ≥ *R_i_*}

The total step count is then derived by calculating the sum of absolute differences between consecutive states over *n* frames:

(2) $$\mathrm{Step}\;\mathrm{Count}=\sum_{i=1}^{n-1}|S_{i}-S_{i-1}|$$

(2)  Duration:

The duration represents the total time elapsed during the walking task [23,24]. It is calculated by dividing the total number of frames (*n*) by the camera’s sampling rate (30 frames per second, FPS). This parameter provides the temporal baseline for calculating higher-level features such as cadence and average velocity. The formula for Duration is defined as:

(3) $$\mathrm{Duration}=\;\frac{n}{30\;\mathrm{FPS}}$$

(3)  Image-plane ankle displacement:

This feature quantifies the pixel-based displacement of the selected ankle landmark between the initial and final frames within the 2D image plane. Because the recordings were obtained using a single uncalibrated smartphone camera, this value should be interpreted as a relative image-plane descriptor of visual landmark progression rather than as a calibrated real-world walking distance. The grounded foot, defined as the ankle with the larger vertical coordinate, was dynamically selected at both the start and end of the sequence to provide a consistent image-plane reference point. The Euclidean distance is then calculated between these two points to reflect the total progression in the 2D plane. The total distance is then calculated using the Euclidean distance formula:

(4) $$\mathrm{Image}-\mathrm{plane}\;\mathrm{ankle}\;\mathrm{displacement}\;=\;\sqrt{{({x_{\mathrm{start}}{-x}_{\mathrm{end}})}^{2}+(y}_{\mathrm{start}}{-y_{\mathrm{end}})}^{2}}$$

(4)  Image-plane mean step displacement:

The Image-plane mean step displacement represents the average spatial displacement covered by a single step [25,26,27]. It is calculated by dividing the total distance by the step count.

(5) $$\mathrm{Image}-\mathrm{plane}\;\mathrm{mean}\;\mathrm{step}\;\mathrm{displacement}=\frac{\mathrm{Vector}\;\mathrm{Distance}}{\mathrm{Step}\;\mathrm{Count}}$$

(5)  Image-plane displacement velocity:

Image-plane displacement velocity was calculated as image-plane ankle displacement divided by walking-segment duration. This variable represents the rate of landmark progression in the two-dimensional image plane under the standardized recording setup. It should not be interpreted as calibrated gait speed or real-world walking velocity because no camera calibration, depth estimation, or physical distance conversion was performed [28,29]. It was calculated as the ratio of image-plane ankle displacement to walking-segment duration. The formula for image-plane displacement velocity is expressed as

(6) $$\mathrm{Image}-\mathrm{plane}\;\mathrm{displacement}\;\mathrm{velocity}=\frac{\mathrm{Vector}\;\mathrm{Distance}}{\mathrm{Duration}}$$

(6)  Mean Step Rate:

The mean step rate is defined as the temporal frequency of steps during the gait cycle. It quantifies the number of occurrences of foot transitions per second, with the unit expressed as s-1 to reflect its nature as a frequency measure. It is calculated by dividing the Step Count by the Duration.

(7) $$\mathrm{Mean}\;\mathrm{Step}\;\mathrm{Rate}=\frac{\mathrm{Step}\;\mathrm{Count}}{\mathrm{Duration}}$$

(7)  Bilateral Mean Gait Stance Cycle:

The Bilateral Gait Cycle was quantified based on the stance of the ankle joints across the sequence. For each frame, the vertical coordinates of the left and right ankle landmarks were compared; the limb exhibiting the larger coordinate value was identified as the lower-positioned foot, signifying the stance phase. The specific frame indices corresponding to these consecutive contact events were recorded independently for the left and right ankles. The duration of each cycle was determined by calculating the frame intervals between successive contact events and converting these counts into seconds by dividing by the acquisition rate. The final features were defined as the mean Left Gait Stance Cycle and the Mean Right Gait Stance Cycle, providing standardized temporal measures of gait periodicity for the classification model.

(8)  Bilateral Stretch Knee angle:

To calculate the Stretch Knee Angle, three landmarks from the MPP are utilized: the hip, the knee, and the ankle. To represent the segments of the lower limb, two vectors are defined for each frame: the thigh vector ($\vec{a}$), originating from the hip and terminating at the knee, and the shank vector ($\vec{b}$), originating from the knee and terminating at the ankle. The knee angle is then derived from the law of cosines of $\vec{a}$ and $\vec{b}$ using the following trigonometric relationship:

$$\mathrm{Stretch}\;\mathrm{Knee}\;\mathrm{Angle}\;=\cos^{-1}\frac{\vec{a}\cdot \vec{b}}{\left|\vec{a}||\vec{b}\right|}$$

To characterize the range of joint motion, the maximum and minimum knee angles are extracted as features from the complete angular time series during the movement.

(9)  Frame Indices of Knee Angles:

This feature was derived by recording the specific frames corresponding to the maximum and minimum knee angles to examine temporal correspondence between estimated 2D knee-angle extrema and gait progression within the image sequence. The maximum angle represents the frame of peak extension, where the lower limb is most stretched, and the minimum angle denotes the frame of peak flexion, characterizing the point of maximum contraction. By synchronizing these angles with the respective frames, the objective was to observe whether a systematic correspondence exists between joint articulation patterns and overall gait progression, thereby identifying potential kinematic deviations between the two groups.

Image-plane ankle displacement, image-plane mean step displacement, and image-plane displacement velocity were expressed in pixel-based units because the gait data were obtained from single-view 2D smartphone video recordings without camera calibration, depth reconstruction, or conversion to real-world units. Therefore, these variables were treated as relative image-plane descriptors rather than absolute biomechanical measures of displacement, stride length, or walking speed. These descriptors may be influenced by camera geometry, participant-to-camera distance, body size, perspective distortion, and device-specific imaging characteristics. Their numerical values may also change with image resolution, cropping, zoom, lens field of view, or changes in the proportion of the image occupied by the participant. The estimated 2D knee-angle descriptors are not expressed in pixels and are less sensitive to uniform image resizing; however, they remain dependent on camera viewpoint, out-of-plane movement, self-occlusion, and landmark-estimation accuracy. Temporal descriptors do not require spatial calibration but depend on an accurate frame rate, temporal segmentation, and reliable step detection. To reduce, but not eliminate, perspective-related variability, the recording setup was standardized, and only gait segments recorded while participants walked toward the camera were analyzed. Formal within-session repeatability, between-session reproducibility, cross-device agreement, and cross-environment reproducibility were not evaluated.

### 2.7. Data Preprocessing and Resampling

To ensure the reliability of the feature extraction process, temporal segmentation and quality-control procedures were applied before model development. A usable gait segment was defined as a temporally cropped toward-camera walking sequence in which the participant walked continuously from the 0 m starting point toward the 5 m endpoint, with sufficient full-body visibility and stable lower-limb landmark detection for at least 3 s. The segment began at the first frame in which the participant initiated forward walking and the body landmarks were sufficiently visible for MediaPipe Pose detection, and ended at the last frame before the participant reached the endpoint, stopped, turned, or initiated return walking. Segments containing turning movements, preparatory movements, post-walking pauses, marked occlusion, incomplete body visibility, unstable lower-limb landmark detection, substantial deviation from the predefined straight path, or usable duration shorter than 3 s were excluded from the final analytical dataset. After temporal segmentation and quality control, the final analytical dataset comprised 404 usable gait segments, including 300 segments from the schizophrenia group and 104 segments from the healthy control group.

In the present study, gait segments were treated as the analytical instances for the feature-comparison and machine-learning analyses. The 404 usable segments were derived from 135 unique participants, and more than one segment from the same participant could be included in the final dataset. Therefore, the 404 segment-level instances should not be interpreted as 404 statistically independent participant-level observations. Repeated segments from the same participant may share participant-specific motor, anatomical, and image-related characteristics, thereby producing within-participant correlation. Because each participant completed three toward-camera traversals, up to 300 candidate segments were available for the schizophrenia group and 105 for the healthy control group. As multiple segments were nested within participants, cluster-aware methods would be required to support participant-level inference. Because such methods were not implemented, the segment-level comparisons were considered exploratory. After quality control, 300 schizophrenia segments and 104 healthy-control segments were retained, corresponding to a mean of 3.00 and 2.97 usable segments per participant, respectively. The exclusion rate among candidate toward-camera segments was therefore 0.0% for the schizophrenia group and 1.0% for the healthy control group.

To address class imbalance in the post-quality-control dataset, the analytical pipeline used for the reported results applied a hybrid resampling strategy to the full post-quality-control dataset before fold partitioning for cross-validation. Thus, class balancing was performed before, rather than exclusively within the training portion of, each cross-validation fold. Specifically, random undersampling was performed on the majority class, and random oversampling with replacement was applied to the minority class until equal class representation was achieved. This procedure resulted in a balanced dataset of 404 instances, with 202 segments in each class. Thus, the resampling procedure changed the class composition of the dataset while maintaining the total number of analytical instances at 404. Because oversampling was performed with replacement before fold partitioning, the same oversampled segment could potentially be represented in both the training and validation folds. In addition, because the analytical unit was the gait segment rather than the participant, multiple segments from the same participant could be represented across different folds. Participant identifiers were not used to constrain fold assignment; consequently, participant-specific motor or image-related characteristics could be represented in both model training and validation. Because resampling preceded segment-wise cross-validation, same-participant and duplicated oversampled segments could occur across training and validation folds, potentially inflating performance. Therefore, these metrics represent exploratory internal comparisons rather than independent participant-level validation; the extent of optimism requires participant-grouped reanalysis. A fixed random seed of 1 was used for both resampling and fold generation to ensure reproducibility.

### 2.8. Machine Learning Models and Cross-Validation

Two machine-learning models, Decision Tree (DT) and Support Vector Machine (SVM), were selected a priori as complementary classifiers for exploratory segment-level group-separation analysis. The machine-learning component was designed as a methodological proof-of-concept rather than as a diagnostic, screening, risk-prediction, or clinical decision-support model. The purpose of applying DT and SVM was to examine whether the extracted image-plane gait descriptors contained internally discriminative information under the present controlled recording and sampling conditions using two structurally different classification approaches.

The DT model was selected because it provides an interpretable nonlinear, rule-based structure that allows visualization of feature hierarchy and decision paths. The SVM model was included as a complementary nonlinear, margin-based classifier for comparison under the same feature set. Thus, the DT represented a single-tree rule-based approach, whereas the SVM provided a non-tree-based linear comparator. These two models were not intended to constitute a comprehensive benchmarking suite or to establish classifier-independent robustness of the 12 gait descriptors. All machine-learning analyses were conducted using Weka software version 3.8.5 (University of Waikato, Hamilton, New Zealand). The DT model was implemented using the J48 algorithm, Weka’s implementation of the C4.5 decision-tree classifier, with a pruning confidence factor of 0.25 and a minimum of two instances per leaf. These parameters were specified to balance model complexity and interpretability. The SVM model was implemented using Weka’s Sequential Minimal Optimization classifier with a polynomial kernel. The polynomial kernel exponent was set to 1.0, making the model functionally equivalent to a linear-kernel SVM under the current implementation. Feature normalization was applied before SVM training to ensure uniform feature scaling during the optimization process [30]. A fixed random seed of 1 was used for both resampling and cross-validation fold generation, and all reported classification metrics were derived from this fixed-seed analysis.

Model performance was assessed using segment-wise 15-fold cross-validation on a segment-level dataset that had already been resampled to balance the class distribution. In each iteration, 14 folds were used for training and the remaining fold was used for validation. Because resampling was performed before fold partitioning and gait segments rather than participants were used as analytical instances, duplicated or non-independent segments could appear across training and validation folds. The folds were not independent at the participant level because no grouping constraint was applied to keep all segments from the same participant within a single fold. Accordingly, the validation folds were not composed exclusively of participants who were unseen during model training. Accordingly, the resulting performance estimates represent potentially optimistic apparent internal segment-level performance under the present analytical design and should not be interpreted as participant-level independent validation, screening performance, diagnostic validity, clinical utility, or readiness for deployment. The models were selected for proof-of-concept comparison rather than comprehensive benchmark optimization. Accordingly, the present analysis cannot determine whether the observed group-separation performance would remain consistent across broader classifier families. Subject-wise cross-validation, grouped k-fold cross-validation, leave-one-subject-out validation, and external validation were not performed in the present analysis. Therefore, the current validation strategy cannot determine whether the trained models would generalize to previously unseen participants. It also cannot provide an unbiased estimate of performance for gait segments obtained from participants who were entirely absent from model training. Accordingly, no subject-independent or participant-level generalization estimate is reported in this study.

### 2.9. Statistical Analysis and Performance Index

Classification performance was summarized using accuracy, precision, recall, F1-score, and Cohen’s Kappa. These performance indices were calculated at the segment level and were not aggregated at the participant level. Therefore, they should be interpreted as internal apparent segment-level performance indices rather than subject-level classification accuracy. These indices were calculated from the confusion matrices generated across the 15 cross-validation iterations and were used to evaluate overall classification performance and agreement beyond chance. All performance metrics were reported as weighted averages based on the instance distribution of each class, thereby providing a summary of model behavior within the resampled segment-level dataset.

Age- and sex-adjusted linear regression models were not included. The healthy control group was substantially younger than the schizophrenia group, and the age distributions of the two groups showed limited overlap. Under these conditions, conventional covariate adjustment would rely heavily on model-based extrapolation beyond the observed common support and could not reliably separate age-related effects from group-related effects. Accordingly, group comparisons of gait features were interpreted as preliminary segment-level differences under the present sampling design rather than as age-independent schizophrenia-associated motor features. Age at illness onset, illness duration, and stable medication status were available for descriptive reporting. However, detailed antipsychotic class, chlorpromazine-equivalent dose, standardized extrapyramidal symptom ratings, standardized psychiatric symptom-severity scores, physical activity measures, and detailed comorbidity data were not systematically available and therefore could not be included as covariates. Consequently, residual confounding related to medication exposure, symptom burden, extrapyramidal manifestations, chronicity, physical activity, and comorbid conditions could not be eliminated in the present analyses.

Group comparisons for the 12 extracted gait features were conducted at the segment level. For each feature, the distributional assumption was evaluated using the Shapiro–Wilk test, and homogeneity of variance was assessed using Levene’s test. When the assumption of equal variances was satisfied, independent-samples t tests with equal variance assumed were applied; otherwise, Welch’s *t* tests were used. Because multiple gait segments were nested within participants, the independence assumption underlying these segment-level tests was not fully satisfied, and the analyses were therefore considered exploratory. Participant-level aggregation and mixed-effects models with participant-level random intercepts and cluster-robust standard errors were not implemented in the present analysis. Thus, the segment-level tests did not explicitly model within-participant correlation, which may have reduced the effective sample size, underestimated standard errors, narrowed confidence intervals, and produced anti-conservative *p* values. The Benjamini–Hochberg FDR procedure addressed multiple testing across the 12 gait features but did not correct for within-participant dependence. The resulting *p* values and confidence intervals may therefore overstate statistical precision and were interpreted as exploratory rather than participant-level inferential evidence. Exact *p* values and FDR-adjusted results were retained for transparency, while interpretation emphasized the direction and magnitude of the group differences rather than statistical significance alone. Effect sizes were quantified using Cohen’s *d* and were interpreted as segment-level descriptive effect sizes rather than participant-level effect estimates.

### 2.10. PyQt5-Based User Interface

**Figure 4:**
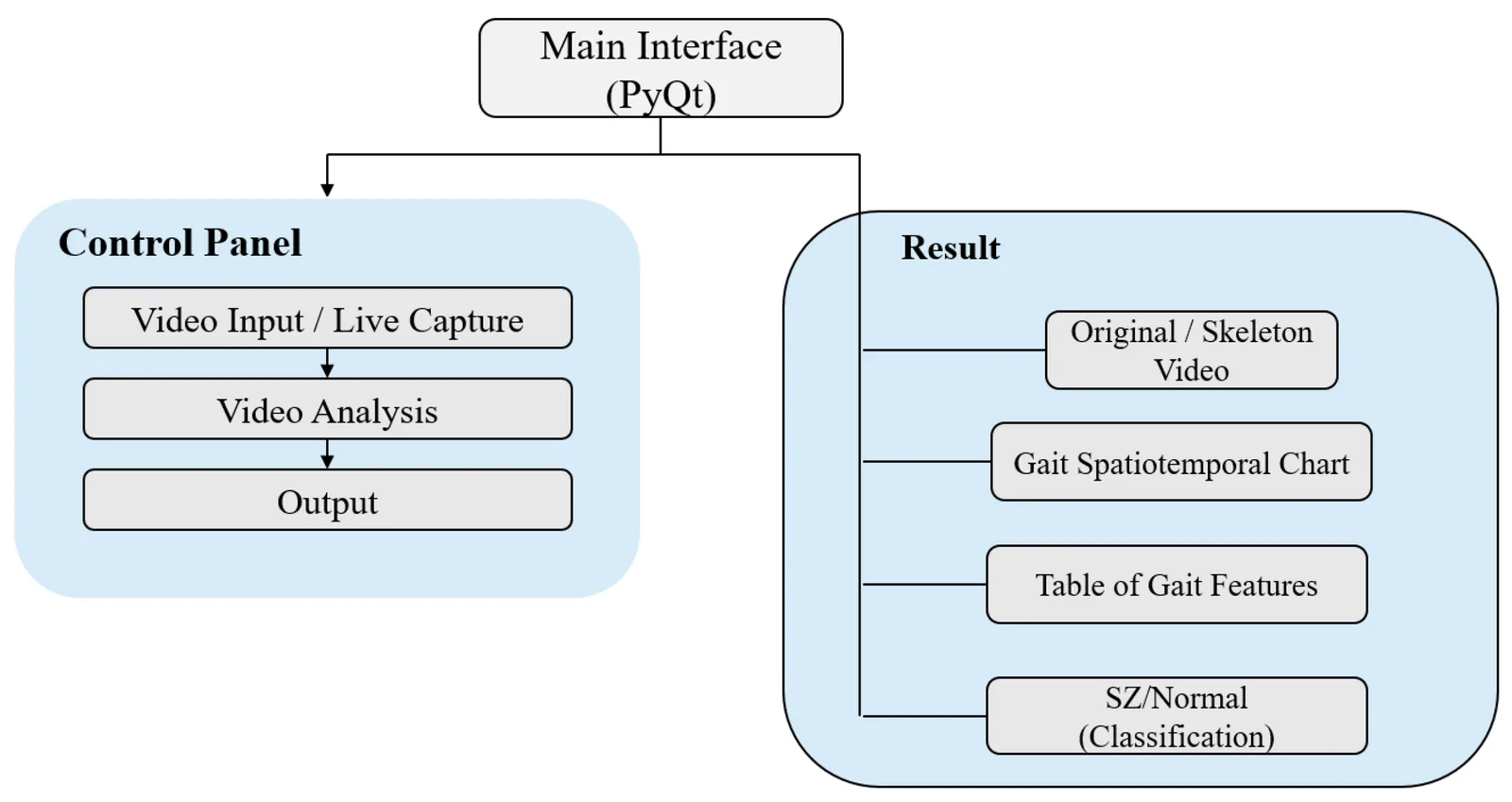
PyQt5-based user interface framework.

**Figure 5:**
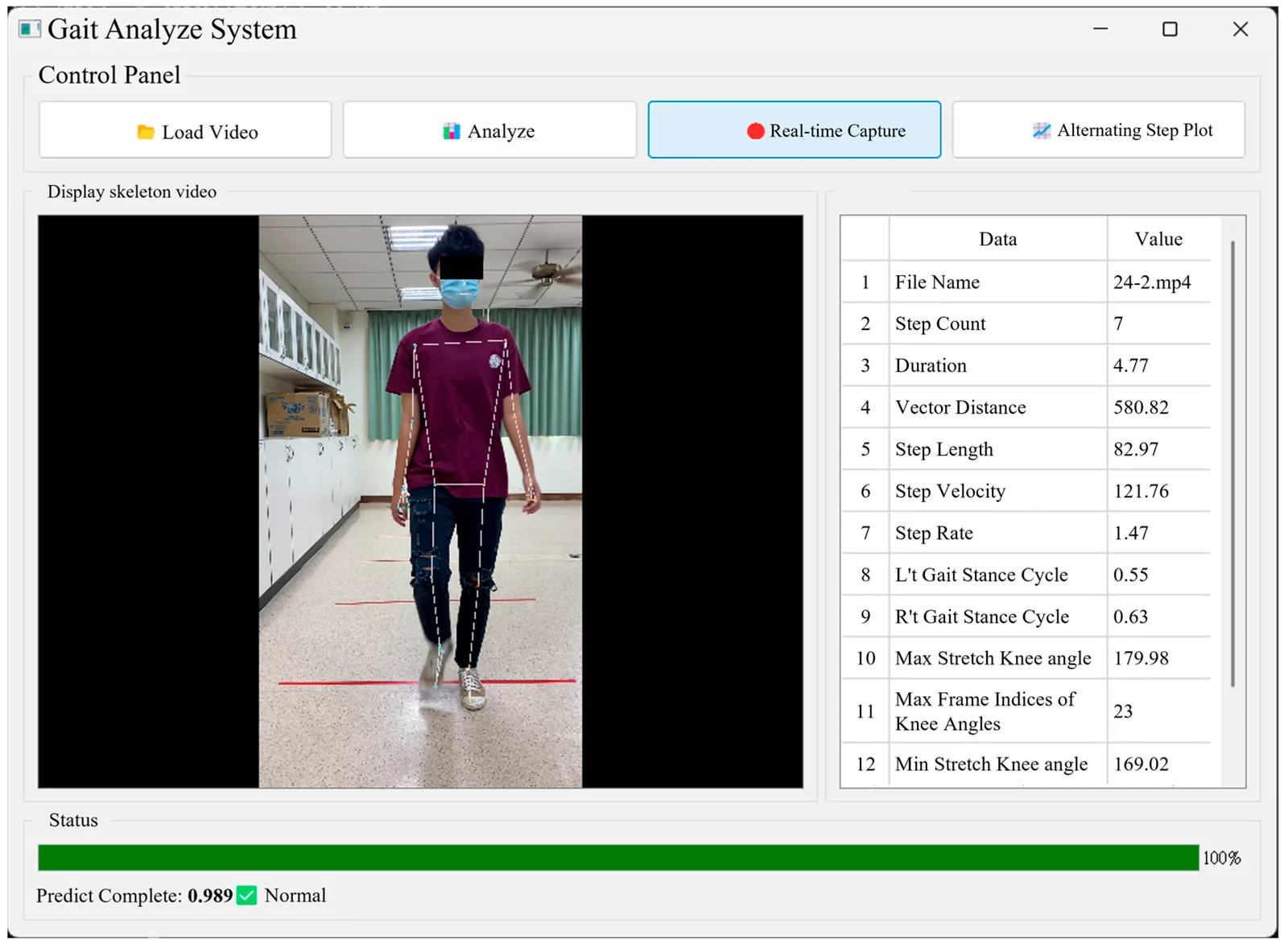
The GUI of the Gait analysis system.

To enhance operational convenience and research translation, the entire framework was integrated into a PyQt5-based user interface, with its architecture illustrated in Figure 4. This research-support visualization interface is designed to display and organize extracted gait features for exploratory analysis by identifying subtle gait signatures. The system offers high flexibility in data acquisition, supporting real-time streaming from mobile or computer cameras as well as the processing of pre-recorded gait videos; the actual system interface is illustrated in Figure 5.

**Figure 6:**
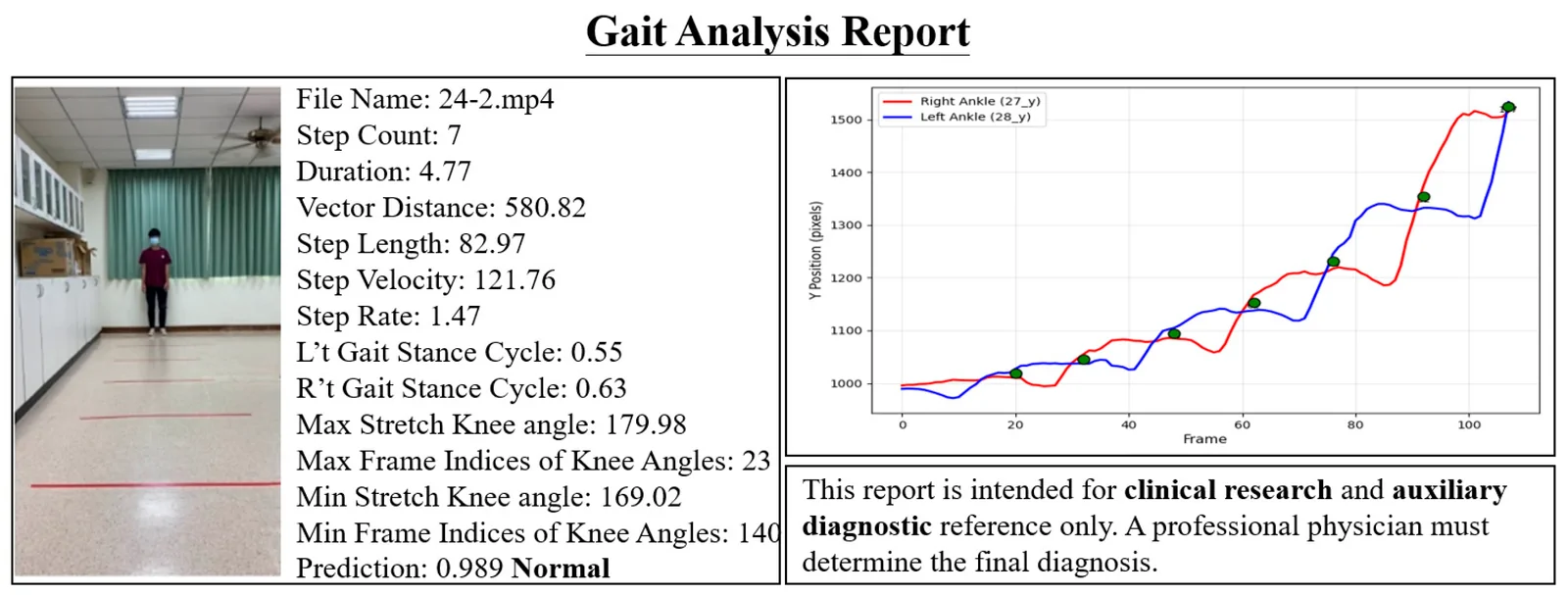
The gait analysis report.

Furthermore, the system features a research-support gait visualization report (Figure 6). This report integrates selected gait-video frames, quantitative image-plane gait descriptors, alternating ankle-trajectory plots, and research-only exploratory segment-level classification outputs. These outputs are intended to summarize internal model behavior and organize extracted gait features for research review. They are not intended to provide diagnosis, screening, risk prediction, treatment recommendation, or clinical decision support. The alternating step plot provides a visual summary of ankle-landmark alternation during walking under the standardized recording setup. For data integrity and subsequent research analysis, the system allows export of extracted feature data for archiving and further analysis.

Characterized by its intuitive design and real-time processing capability, the system provides a research-support tool for visualizing and summarizing extracted gait features under standardized recording conditions for approved research use. By integrating image-plane gait-feature extraction with a structured visualization interface, the system illustrates the potential utility of AI-assisted methods for exploratory psychiatric motor research.

## 3. Results

Table 2 summarizes the demographic and clinical characteristics of the study participants. The schizophrenia group included 100 patients, whereas the healthy control group included 35 participants. The schizophrenia group had a mean age of 47.82 ± 11.01 years, while the healthy control group had a mean age of 21.85 ± 0.54 years. The corresponding between-group difference in mean age was 25.97 years, indicating a pronounced age imbalance between the enrolled groups. The markedly smaller standard deviation in the healthy control group also indicates that the control participants were concentrated within a narrow, substantially younger age range. Sex distributions were 51.0% male and 49.0% female in the schizophrenia group, and 42.9% male and 57.1% female in the healthy control group. In the schizophrenia group, the mean age at illness onset was 24.00 ± 8.01 years and the mean illness duration was 22.63 ± 11.50 years. All patients with schizophrenia had received stable medication during the month prior to data collection. However, detailed antipsychotic class, chlorpromazine-equivalent dose, duration of antipsychotic exposure, standardized extrapyramidal symptom ratings, standardized psychiatric symptom-severity scores, physical activity measures, and detailed information regarding relevant comorbidities were not systematically available. These variables could therefore neither be summarized reliably nor incorporated into adjusted analyses. The healthy control group was not age-matched to the schizophrenia group; therefore, age-related gait differences could not be separated from schizophrenia-associated motor features in the present design. The age imbalance may also have contributed to the apparent classification performance because age was strongly associated with group membership and is known to affect gait speed, step characteristics, balance control, and lower-limb kinematics [31,32]. Accordingly, the following segment-level group comparisons should be interpreted as differences between the enrolled schizophrenia group and the younger healthy control group, rather than as age-independent schizophrenia-associated gait differences. Because no psychiatric comparison group was included, these analyses cannot determine whether the observed gait-feature differences are specific to schizophrenia or reflect broader psychiatric, medication-related, lifestyle-related, or general motor-function differences. Moreover, the observed gait-feature differences cannot be attributed directly to schizophrenia because the potential contributions of antipsychotic exposure, extrapyramidal manifestations, psychiatric symptom burden, illness chronicity, reduced physical activity, and relevant comorbidities could not be separated within the current dataset.

Table 3 presents the segment-level comparisons of the 12 extracted gait features between the schizophrenia and healthy control groups. For each feature, normality and homogeneity-of-variance assumptions were evaluated before hypothesis testing. When the equal-variance assumption was not satisfied, Welch’s t test was used instead of the standard independent-samples *t* test. To account for multiple testing across the 12 features, *p* values were adjusted using the Benjamini–Hochberg false discovery rate (FDR) procedure. The reported 95% confidence intervals represent the confidence intervals of the mean difference between groups. Under the present non-age-matched sampling design, the exploratory segment-level comparisons suggested group-difference patterns in several image-plane spatiotemporal and estimated 2D knee-kinematic descriptors between the enrolled schizophrenia group and the younger healthy control group. Descriptively, the younger healthy control group showed higher values than the schizophrenia group for Step Count, Duration, Image-plane ankle displacement, Image-plane mean step displacement, and Image-plane displacement velocity, as shown in Table 3. These findings are consistent with greater image-plane displacement, longer image-plane mean step displacement, and higher image-plane displacement velocity under the present recording conditions. Descriptively, Image-plane ankle displacement (d = −1.726) and Minimum Knee Angle Frame (d = −1.042) showed the largest segment-level effect sizes. In addition, Maximum Stretch Knee Angle and Minimum Stretch Knee Angle differed between groups, consistent with group differences in estimated 2D knee-angle descriptors under the present image-based recording conditions.

Because multiple gait segments were obtained from the same participants, the segment-level observations were not fully independent. Therefore, the independence assumption underlying the segment-level t tests was not fully satisfied. The FDR correction addressed multiple comparisons across features but did not account for within-participant clustering; consequently, the *p* values may be anti-conservative and the confidence intervals may be narrower than those obtained from a cluster-aware analysis. The magnitude of this potential bias cannot be determined without cluster-aware reanalysis. These analyses were retained to describe segment-level group-difference patterns within the current dataset, but they should not be interpreted as participant-level statistical inference. Interpretation therefore emphasizes the direction and magnitude of the observed differences, particularly descriptive mean differences and Cohen’s d which represent segment-level descriptive estimates. No participant-level statistical significance is claimed.

All reported model-performance indices were obtained from the fixed-seed analysis using random seed = 1. These indices were used to describe exploratory internal group-separation behavior within the resampled segment-level dataset and were not intended to represent diagnostic accuracy, screening performance, or clinical decision-support validity. Under the present pre-cross-validation resampling and segment-wise 15-fold cross-validation design, the DT model showed higher apparent internal segment-level performance than the SVM among the two prespecified classifiers, achieving an accuracy of 98.52% and a Kappa value of 0.970. Because class balancing preceded fold allocation and the folds were not grouped by participant, these values may be optimistic because same-participant and duplicated oversampled segments could occur across training and validation folds.

As shown in Table 4, the weighted-average precision, recall, and F1-score were all 0.985. At the class level, the healthy control group showed a precision of 0.990 and a recall of 0.980, whereas the schizophrenia group showed a precision of 0.980 and a recall of 0.990. Accordingly, the remarkably high 98.52% accuracy represents potentially optimistic apparent internal segment-level performance within the current resampled dataset. It neither estimates performance in previously unseen participants nor establishes participant-level diagnostic or screening validity, clinical utility, or readiness for deployment.

**Figure 7:**
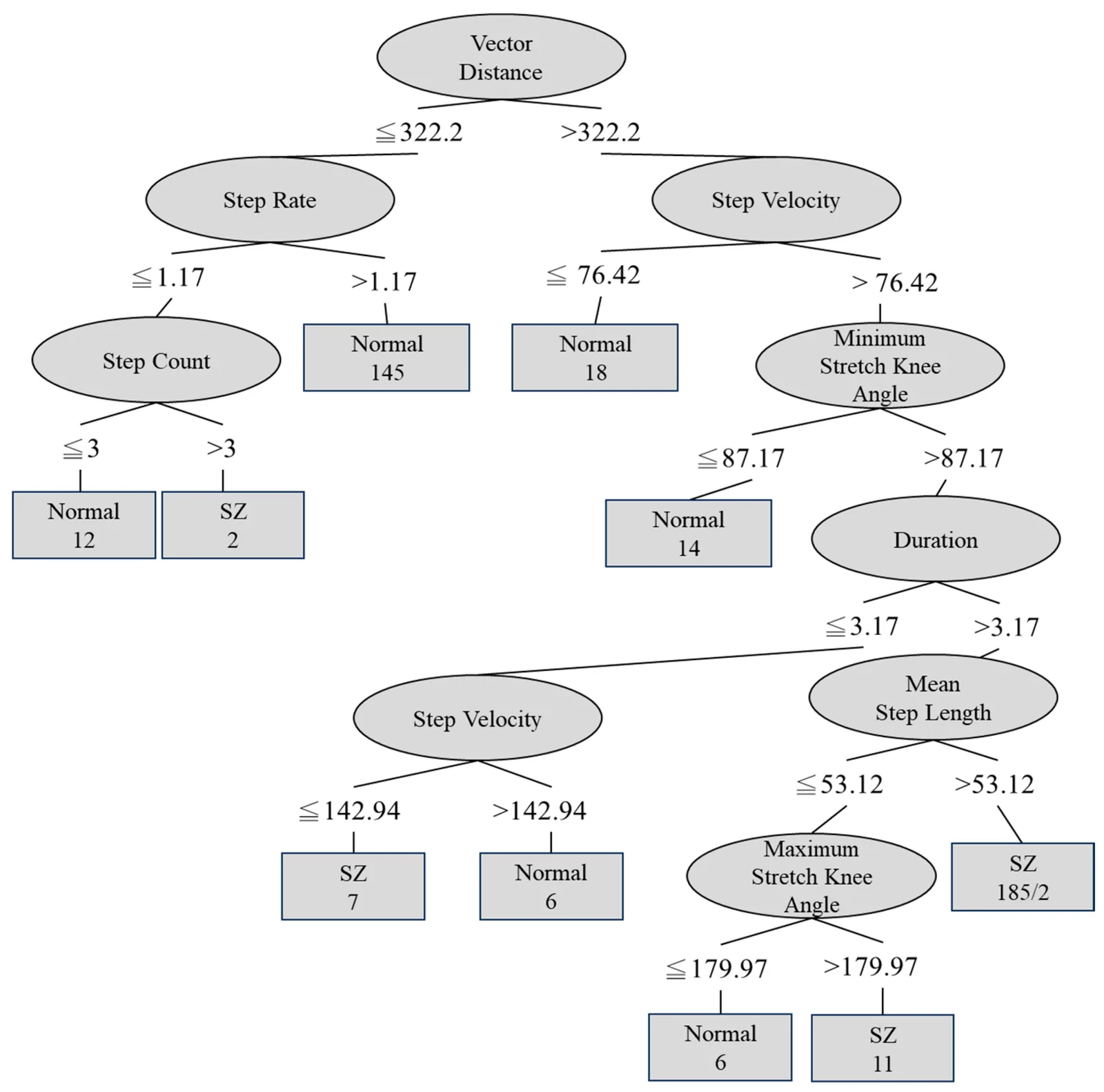
Hierarchical structure of DT.

The hierarchical structure of the DT model is shown in Figure 7. The DT hierarchy should be interpreted as an exploratory description of internal model behavior within the current segment-level dataset, not as a clinical decision rule or participant-level diagnostic algorithm. Image-plane ankle displacement served as the root node, indicating that it was the most influential feature in the initial split between the schizophrenia and healthy control gait segments. Because this feature was derived from pixel-based image-plane coordinates, its role as the root node should be interpreted as indicating high discriminative value within the present controlled recording setup, rather than as evidence that calibrated ankle displacement or real-world gait distance was the primary biomechanical determinant of group separation. For instances with Image-plane ankle displacement ≤ 322.2, pixels under the present fixed camera configuration, the model further split the data using Step Rate and Step Count. For instances with Image-plane ankle displacement > 322.2, pixels under the same configuration, the classification hierarchy incorporated Image-plane displacement velocity, Estimated minimum 2D knee angle, Duration, Image-plane mean step displacement, and Estimated maximum 2D knee angle. These pixel-based split values are dataset- and configuration-specific and were not evaluated as transferable thresholds across devices, resolutions, or recording environments. Overall, this branching structure indicates how the internally trained DT model organized the selected image-plane and estimated 2D gait descriptors for segment-level group separation within the current dataset.

Under the same pre-cross-validation resampling and segment-wise 15-fold cross-validation design, the SVM model also demonstrated apparent internal segment-level group-separation performance within the current dataset, although its overall performance was lower than that of the DT model. The SVM achieved an accuracy of 89.7% and a Kappa value of 0.793. These estimates are subject to the same potential participant-overlap and duplicated-segment leakage as the DT results and therefore do not represent subject-independent validation. As shown in Table 5, the weighted-average precision, recall, and F1-score were all 0.897. At the class level, the healthy control group showed a precision of 0.893, a recall of 0.901, and a F1-score of 0.897, whereas the schizophrenia group showed a precision of 0.900, a recall of 0.892, and a F1-score of 0.896. As with the DT results, these values should be interpreted as performance estimates obtained under the current analytical framework rather than as participant-level independent screening accuracy. As with the DT results, the SVM performance estimates should be considered research-only exploratory internal segment-level group-separation results rather than participant-level validation, diagnostic classification, screening performance, or clinical decision-support evidence.

To examine feature contribution within the SVM framework, we reviewed the attribute weights derived from the model. The five highest-weighted features were Image-plane ankle displacement, Minimum Stretch Knee Angle, Image-plane displacement velocity, Image-plane mean step displacement, and Step Count. Compared with the DT hierarchy, which emphasized a sequential split structure led by Image-plane ankle displacement, the SVM results suggest a broader contribution from both spatiotemporal and joint-kinematic features. However, because the present study treated the DT hierarchy as the primary interpretability analysis, the SVM findings should be regarded mainly as a complementary feature-contribution pattern rather than as a fully equivalent interpretability model.

## 4. Discussion

**Figure 8:**
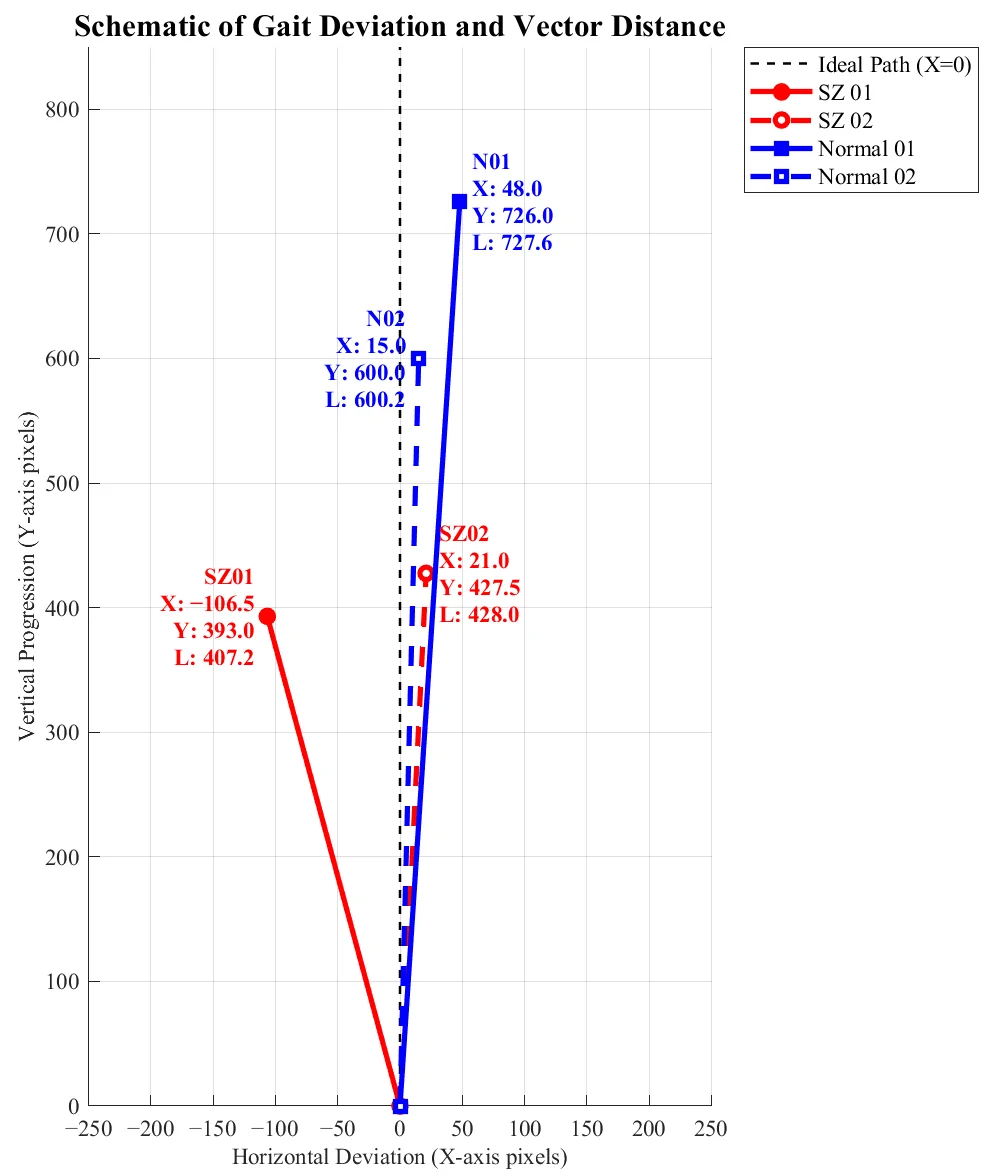
Schematic of Gait Deviation and Image-plane ankle displacement.

In this study, both the DT and SVM models identified Image-plane ankle displacement as an influential discriminative feature under the present sampling and validation design. In the DT model, Image-plane ankle displacement served as the root node of the classification hierarchy, indicating that it provided the primary split between gait segments from the enrolled schizophrenia group and those from the younger healthy control group. This feature hierarchy should be interpreted as the split structure of the internally trained segment-level DT model and may not remain stable under subject-wise validation, external validation, or participant-level aggregation. Although all participants walked along the same standardized 5 m path, the schizophrenia group showed a significantly shorter image-plane ankle displacement than the healthy control group. This pattern may reflect lower image-plane landmark progression and greater apparent image-plane lateral offset under the present recording conditions. However, these image-plane descriptors cannot be equated with calibrated forward displacement, mediolateral sway, or biomechanical gait efficiency because single-camera and markerless gait measures are sensitive to camera geometry, scaling procedures, viewpoint, occlusion, and validation methods [14,15,16,20,21,22]. Similarly, because detailed medication exposure, extrapyramidal symptoms, psychiatric symptom severity, physical activity level, and comorbidity data were unavailable, the observed image-plane gait differences cannot be interpreted as medication-independent motor abnormalities or as direct evidence of primary schizophrenia-related motor dysfunction [1,2,3,4,5,6,7]. Figure 8 schematically illustrates the observed gait-deviation pattern and the image-plane ankle-displacement descriptor under the standardized recording configuration.

At the next level of the classification hierarchy, Step Rate and Image-plane displacement velocity further contributed to group differentiation in the DT model. Under the present short-distance protocol, the schizophrenia group showed a gait pattern that was relatively frequent but slow, suggesting that increased step count over a limited walking distance may have contributed to a higher calculated cadence despite lower image-plane displacement per walking segment. This observation partially differs from the previous literature, which has more commonly linked reduced walking speed in schizophrenia or psychosis to shortened step length rather than increased cadence [2,6,7]. In contrast, the SVM placed greater emphasis on Estimated minimum 2D knee angle in addition to Image-plane displacement velocity, suggesting that the two models captured partially different aspects of gait abnormality. Whereas the DT primarily emphasized spatiotemporal organization, the SVM appeared to reflect a combination of spatiotemporal and localized joint-kinematic variation. Taken together, these findings suggest that, within the present dataset, gait-feature differences between the enrolled schizophrenia group and the younger healthy control group involved both image-plane landmark-progression descriptors and estimated 2D lower-limb kinematic descriptors. However, the clinical sources of these differences remain indeterminate because medication, extrapyramidal symptom, psychiatric symptom severity, physical activity, and comorbidity data were unavailable [1,2,3,4,5,6,7].

Motor dysfunction is common in schizophrenia and has been discussed as a clinically relevant but underrepresented domain in psychiatric assessment and rehabilitation-oriented monitoring [1,2,3,4,5]. Against this background, the present study provides a quantitative framework for examining gait characteristics observed in schizophrenia samples under controlled recording conditions. By integrating MPP-based coordinate extraction, 12 derived gait features, and a centralized GUI, the system offers a structured workflow for quantitative gait analysis. Accordingly, the proposed framework should be interpreted as a proof-of-concept and research-support tool for exploratory gait assessment rather than as a standalone diagnostic or screening system. The classification component should be interpreted as a methodological analysis of internal group separation rather than as a patient-level predictive model. The GUI-based output is therefore best understood as a research visualization and feature-organization interface, not as a clinical decision-support system.

The sensitivity to acquisition conditions also differs across feature types. Pixel-based spatial descriptors are directly scale- and configuration-dependent. Estimated 2D knee angles are less affected by uniform image resizing but remain viewpoint-dependent and may be altered by out-of-plane movement, occlusion, and landmark-estimation error. Temporal descriptors are not dependent on spatial calibration but require an accurate frame rate and reproducible temporal segmentation and step detection. Formal robustness testing, within-session or between-session repeatability analysis, cross-device agreement, and cross-site reproducibility assessment were not performed. Therefore, the present findings should be interpreted as evidence of controlled-setting feasibility rather than proof of cross-device or cross-environment portability. Future studies should separately evaluate repeatability under an unchanged configuration, robustness under systematically varied camera and environmental conditions, and reproducibility across devices and sites.

A staged reproducibility evaluation is therefore required. First, repeated recordings obtained without changing the acquisition configuration should be used to quantify within-session and between-session repeatability using intraclass correlation coefficients, within-subject coefficients of variation, standard errors of measurement, minimal detectable change, and Bland–Altman analysis. Second, robustness should be evaluated by systematically varying camera model, image resolution, frame rate, camera height, camera-to-participant distance, pitch, yaw, lens field of view, lighting, background, and operator while the underlying walking task is held constant. Third, cross-site reproducibility should be examined using a prespecified acquisition protocol and independent participant cohorts.

A corrected validation analysis should use participant-grouped (subject-wise) validation strategies, such as grouped k-fold cross-validation or leave-one-subject-out validation, in which all segments from the same participant are assigned exclusively to either the training or validation set within each fold. Resampling, feature scaling, feature selection, and hyperparameter tuning should be conducted exclusively within the training portion of each fold, followed by evaluation on an untouched participant-level validation fold. Performance should then be calculated from participant-independent out-of-fold predictions rather than from folds containing overlapping participant identities. External validation should subsequently be performed in an independent participant cohort. Before clinical applicability can be considered, the framework also requires prospective patient-level evaluation in age-matched and clinically relevant psychiatric comparison cohorts, with prespecified outcomes and assessment of diagnostic specificity and clinical utility. Participant-level aggregation or mixed-effects modeling with participant-level random effects, or cluster-robust statistical procedures, should also be considered to account for within-subject clustering.

This study also has several limitations related to model comparison and interpretability. The comparison was restricted to DT and SVM, without additional baseline models such as Logistic Regression, Random Forest, or XGBoost. Although DT and SVM represent structurally different single-tree and linear margin-based approaches, comparison of only these two classifiers does not establish that the discriminative contribution of the 12 gait descriptors is independent of model architecture. Therefore, classifier-independent feature robustness remains unestablished. Because the present validation strategy was not subject-wise, differences between DT and SVM performance should be interpreted as model behavior within the current resampled segment-level dataset rather than as definitive evidence that one algorithm generalizes better to new participants. Hyperparameters were pre-specified rather than systematically optimized through grid search, nested cross-validation, or other tuning procedures. Interpretability was emphasized mainly through the DT split hierarchy, which directly reveals how the classifier prioritizes the extracted gait features. In contrast, the SVM was treated primarily as a comparative classifier rather than as a fully interpretable feature-ranking model, and no additional post hoc explainability analyses, such as permutation importance, LIME, or SHAP, were performed [33]. Therefore, the current findings should be interpreted as an exploratory model comparison within a proof-of-concept framework.

Recent AI-assisted human-motion research illustrates a broader methodological approach. Although Glove-Net addressed grasp classification rather than gait analysis, it compared conventional machine-learning and deep-learning architectures, evaluated posture-only, force-only, and fused multisensory inputs, and used ablation experiments to assess model and modality contributions [34]. This framework highlights the value of testing multiple classifier families under common evaluation conditions and quantifying the incremental contribution of complementary movement signals. However, direct comparison of accuracy values would be inappropriate because the research tasks, samples, sensing modalities, and validation designs differ. The present study used only video-derived gait descriptors, compared only DT and SVM, and did not conduct multimodal fusion or ablation analysis. Glove-Net therefore informs future methodological development rather than directly validating the present performance estimates.

Future studies should incorporate broader baseline benchmarking, including Logistic Regression, Random Forest, and boosting-based classifiers such as XGBoost, systematic hyperparameter tuning, more formal explainability analyses, predefined feature-family ablation analyses, multimodal fusion when synchronized complementary signals are available, and better-matched comparison groups to further clarify the robustness and interpretability of candidate gait-related motor features in schizophrenia research. Within the present framework, ablation analyses could compare spatiotemporal-only, estimated 2D joint-kinematic-only, and combined feature sets to quantify their incremental contributions. When an adequate participant-level sample and temporally resolved inputs are available, sequence-aware deep-learning models may also be compared with conventional classifiers under the same participant-grouped validation framework. All classifier, feature-set, and modality comparisons should use identical participant-grouped data partitions, with preprocessing, resampling, feature selection, and hyperparameter optimization conducted exclusively within the training data, followed by evaluation in an independent external cohort. In addition, the healthy control group consisted of third-year undergraduate university students and was screened for psychiatric history based only on self-report rather than structured diagnostic assessment. This recruitment strategy may have introduced selection bias and limits the comparability between the schizophrenia and control groups. Future work should use structured psychiatric screening for control participants and should recruit comparison groups that are matched by age, sex, body size, physical activity level, and relevant clinical characteristics.

Video-based gait analysis may also be affected by differential segment retention. If one group has a higher proportion of excluded videos or unusable segments, the final analytical dataset may overrepresent participants whose gait could be more easily captured and tracked, thereby introducing selection bias. In the present study, the exclusion rate among candidate toward-camera segments was low; however, future studies should prospectively document video-level exclusion reasons and examine whether segment retention varies by diagnostic group, motor severity, or recording quality. This is particularly important because video-based pose-estimation algorithms may be affected by occlusion, viewpoint, clothing, camera placement, and movement characteristics [14,15,16,20,21,22].

The present findings should be interpreted within clearly defined clinical and ethical boundaries. This framework is not intended to diagnose schizophrenia, replace psychiatric assessment, or infer mental health status from gait data in non-consenting individuals. In particular, the exploratory classification outputs should not be used to label individuals, estimate psychiatric risk, guide clinical decisions, or infer mental health status outside the consented research context. Rather, it was developed as a research-support tool for quantifying gait-related motor phenotypes among individuals with schizophrenia. Because gait videos may reveal sensitive health-related and potentially identifiable movement information, future implementation should incorporate explicit informed consent, restricted access to raw videos, de-identification procedures, secure storage, transparent data governance, and clear prohibition of non-consented use in employment, insurance, legal, educational, or surveillance contexts [35]. These safeguards are particularly important for AI-assisted analysis of health-related data, where risks include inappropriate secondary use, algorithmic overinterpretation, and deployment beyond the conditions under which the model was developed [35].

A major limitation is the absence of psychiatric comparison groups. The current comparison between individuals with schizophrenia and healthy controls cannot determine whether the observed gait features are specific to schizophrenia, reflect transdiagnostic motor impairment, medication-related effects, chronic institutional or lifestyle factors, reduced physical activity, or broader psychiatric disability. Therefore, the diagnostic specificity of the observed image-plane gait-feature differences remains untested. This limitation was further compounded by the absence of medication-detail and symptom-severity variables within the schizophrenia group, which prevented within-group examination of whether gait features varied according to antipsychotic exposure, extrapyramidal symptoms, negative symptoms, or illness severity [1,2,3,4,5,6,7]. Future studies should include participants with mood disorders, other psychotic disorders, medication-induced extrapyramidal symptoms, neurological conditions, and musculoskeletal conditions to evaluate diagnostic specificity and clinical utility.

### Limitations

Several limitations should be considered when interpreting the present findings. First, the schizophrenia and healthy control groups were not age-matched. The mean age was 47.82 ± 11.01 years in the schizophrenia group and 21.85 ± 0.54 years in the healthy control group, with limited overlap between their age distributions. The healthy controls were undergraduate university students and were screened for psychiatric history by self-report rather than structured diagnostic assessment. In addition, psychiatric comparison groups were not included. Therefore, the observed gait-feature differences and apparent classification performance may partly reflect age, recruitment, lifestyle, or broader diagnostic differences and cannot be interpreted as age-independent or schizophrenia-specific effects.

Detailed antipsychotic exposure, chlorpromazine-equivalent dose, extrapyramidal symptom ratings, psychiatric symptom-severity measures, physical activity, and relevant comorbidity data were also unavailable. Their contributions could not be separated from schizophrenia-associated motor phenomena within the current design. Future studies should recruit age-, sex-, and body-size-matched controls, include clinically relevant psychiatric comparison groups, use structured diagnostic screening, and prospectively collect detailed medication, symptom, physical activity, and comorbidity data.

Second, the spatial gait descriptors were derived from uncalibrated single-view image-plane coordinates. Camera placement, walking distance, and recording orientation were standardized, but no physical reference length, camera calibration, depth reconstruction, body-size normalization, or conversion to real-world units was performed. The spatial descriptors and Decision Tree thresholds should therefore be interpreted as configuration-specific relative image-plane measures rather than calibrated estimates of stride length, walking speed, displacement, or three-dimensional biomechanics. Formal within- and between-session repeatability, cross-device agreement, and cross-site reproducibility were not evaluated. Future validation should incorporate calibrated physical references or floor-plane transformation, camera calibration or depth sensing, body- or image-based normalization where appropriate, and comparison with laboratory-based motion capture or another gold-standard gait-analysis system [14,15,16,20,21,22].

Third, the statistical analyses were performed at the segment level. The 404 segments were obtained from 135 participants and did not constitute 404 independent biological observations. Participant-level aggregation, mixed-effects models, and cluster-robust procedures were not implemented; consequently, the segment-level *t* tests and Welch’s t tests did not account for within-participant correlation. The reported standard errors may therefore be underestimated, the confidence intervals may be too narrow, and the *p* values may be anti-conservative. The FDR correction addressed multiplicity across gait features but did not resolve within-participant dependence. Accordingly, these results should be interpreted as exploratory segment-level descriptive patterns rather than participant-level statistical inference. Confirmatory analyses should use linear mixed-effects models, participant-level aggregation, or cluster-robust procedures, followed by multiplicity correction of the cluster-aware group-effect tests [36,37,38,39].

Fourth, the machine-learning validation was segment-wise rather than participant-grouped, and the full segment-level dataset was resampled before fold allocation. Segments from the same participant—and potentially duplicated oversampled segments—could therefore occur in both training and validation folds. The reported classification metrics may consequently reflect participant overlap and duplicated-segment leakage and should be interpreted only as potentially optimistic apparent internal segment-level performance. They do not establish generalization to previously unseen participants, diagnostic or screening validity, clinical utility, or readiness for deployment. Future studies should use participant-grouped cross-validation, with resampling, preprocessing, feature selection, and hyperparameter tuning confined to the training folds, followed by independent external and prospective clinical validation.

Finally, model comparison was restricted to DT and SVM, hyperparameters were prespecified, and broader classifier benchmarking, multimodal fusion, predefined feature-family ablation, and post hoc explainability analyses were not performed. Differential segment retention and the low but potentially group-dependent exclusion of unusable recordings may also introduce selection bias. Future research should compare multiple classifier families under identical participant-grouped partitions, quantify the incremental contributions of different feature families and complementary sensing modalities, systematically document exclusion reasons, and evaluate performance in independent and clinically representative cohorts. These limitations collectively support interpreting the present framework as a controlled-setting proof of concept rather than a participant-level predictive or clinically deployable system.

## 5. Conclusions

This study presents a computer-vision-based framework for quantifying controlled-setting gait-feature differences between individuals with clinically diagnosed schizophrenia and healthy controls. The methodology transforms raw smartphone video data into 12 image-plane spatiotemporal and estimated 2D knee-kinematic descriptors using MPP-based coordinate extraction. Within the current dataset, both DT and SVM identified Image-plane ankle displacement as one of the most influential discriminative features, although these findings should be interpreted within the exploratory design of the study.

This study demonstrates the feasibility of a markerless, smartphone-based video framework for quantifying gait-related motor phenotypes in individuals with clinically diagnosed schizophrenia under a single controlled camera configuration. However, the non-age-matched cohort, residual clinical confounding, uncalibrated image-plane measurements, segment-level dependence, and non-participant-grouped validation substantially limit participant-level, disease-specific, biomechanical, and clinical inference. The statistical and machine-learning findings should therefore be interpreted as exploratory segment-level results obtained within the current controlled dataset. Future studies require appropriately matched and clinically relevant comparison groups, cluster-aware statistical analysis, participant-grouped and external validation, calibrated biomechanical measurement, and systematic reproducibility evaluation before clinical translation can be considered.

## Figures and Tables

**Table 1:** 12 Gait Features.

Features Name	Unit	Description
Step Count	Counts	Total number of detected steps
Duration	Seconds	Video frame count divided by frame rate (30 FPS) converted into seconds
Image-plane ankle displacement	Pixel	Pixel-based Euclidean displacement of ankle landmarks between the initial and final frames in the 2D image plane
Image-plane mean step displacement	Pixel/Step	Pixel-based image-plane ankle displacement divided by detected step count
Image-plane displacement velocity	Pixel/s	Pixel-based image-plane displacement divided by walking-segment duration
Step Rate	Step/s	Number of steps per second
Estimated left stance-cycle interval	Seconds	Time required for one complete swing cycle of the left foot
Estimated right stance-cycle interval	Seconds	Time required for one complete swing cycle of the right foot
Estimated maximum 2D knee angle	Degree (°)	Maximum extension angle of the knee joint during movement
Estimated minimum 2D knee angle	Degree (°)	Minimum extension angle of the knee joint during movement
Maximum Knee angle Frame	Number of Frame	Video frame corresponding to the maximum knee angle
Minimum Knee angle Frame	Number of Frame	Video frame corresponding to the minimum knee angle

**Table 2:** Demographic and clinical characteristics of the participants.

Group	*n*	Age, Mean ± SD	Sex, Male/Female, *n* (%)	Height, Mean ± SD	Weight, Mean ± SD	BMI, Mean ± SD	Age of Onset, Mean ± SD	Illness Duration, Mean ± SD	Stable Medication During the Previous Month, *n* (%)
Patients with schizophrenia	100	47.82 ± 11.01	51 (51.0)/49 (49.0)	163.73 ± 8.87	68.58 ± 16.20	25.49 ± 5.28	24.00 ± 8.01	22.63 ± 11.50	100 (100%)
Healthy controls	35	21.85 ± 0.54	15 (42.9)/20 (57.1)	165.16 ± 9.63	69.73 ± 13.21	25.50 ± 3.92	NA	NA	NA

**Table 3:** Exploratory segment-level comparisons of the 12 extracted gait features between the schizophrenia and healthy control groups.

Features	Mean ± SD		95% CI ^1^		t	*p*	Cohen’s *d*
	SZ (N = 300)	Healthy Control (N = 104)	LL	UL			
Step Count	5.40 ± 2.76	7.13 ± 1.25	−2.132	−1.342	−8.646	<0.001 ***	−0.702
Duration	3.32 ± 2.26	4.38 ± 0.93	−1.376	−0.750	−6.680	<0.001 ***	−0.528
Image-plane ankle displacement	292.62 ± 152.56	575.63 ± 193.41	−319.650	−246.361	−15.182	<0.001 ***	−1.726
Image-plane mean step displacement	56.45 ± 35.11	82.84 ± 37.10	−34.350	−18.423	−6.514	<0.001 ***	−0.741
Image-plane displacement velocity	100.49 ± 52.62	132.16 ± 33.05	−42.492	−20.859	−5.757	<0.001 ***	−0.654
Step Rate	1.78 ± 0.57	1.67 ± 0.28	0.032	0.201	2.711	0.007 **	−0.215
Estimated left stance-cycle interval	0.50 ± 0.14	0.52 ± 0.07	−0.035	0.008	−1.202	0.230	−0.159
Estimated right stance-cycle interval	0.52 ± 0.15	0.53 ± 0.79	−0.037	0.010	−1.121	0.263	−0.024
Estimated maximum 2D knee angle	178.46 ± 9.52	179.93 ± 0.11	−2.543	−0.386	−2.673	0.008 **	−0.179
Maximum knee-angle frame	48.69 ± 56.43	58.61 ± 37.35	−21.592	1.758	−1.670	0.096	−0.190
Estimated minimum 2D knee angle	146.07 ± 42.66	162.44 ± 8.32	−21.456	−11.279	−6.326	<0.001 ***	−0.442
Minimum knee-angle frame	47.36 ± 48.02	96.64 ± 45.04	−57.855	−36.718	−8.796	<0.001 ***	−1.042

^1^ The 95% CI refers to the confidence interval of the segment-level mean difference between groups. *p* values shown in this table are Benjamini–Hochberg FDR-adjusted *p* values. Because multiple segments could be obtained from the same participant, neither the *p* values nor the confidence intervals account for within-participant clustering. These statistics should therefore be interpreted as exploratory segment-level estimates rather than participant-level inference. ** *p* < 0.01; *** *p* < 0.001. The significance symbols refer to Benjamini–Hochberg FDR-adjusted *p* values.

**Table 4:** Apparent internal segment-level performance metrics of the Decision Tree under pre-cross-validation resampling and segment-wise 15-fold cross-validation.

Class	TP Rate	FP Rate	Precision	Recall	F1 Score
Healthy control	0.980	0.010	0.990	0.980	0.985
Schizophrenia	0.990	0.020	0.980	0.990	0.985
Weighted Avg.	0.985	0.015	0.985	0.985	0.985

Note: These metrics were calculated after balancing the full segment-level dataset before fold allocation and without participant-grouped validation. They may therefore be optimistic and should not be interpreted as estimates of performance in previously unseen participants, participant-level diagnostic or screening accuracy, or clinical utility.

**Table 5:** Performance metrics of the Support Vector Machine.

Class	TP Rate	FP Rate	Precision	Recall	F1 Score
Healthy control	0.901	0.108	0.893	0.901	0.897
Schizophrenia	0.892	0.099	0.900	0.892	0.896
Weighted Avg.	0.897	0.103	0.897	0.897	0.897

## Data Availability

The de-identified derived gait-feature data supporting the findings of this study may be made available by the corresponding author upon reasonable request, subject to institutional approval, execution of a data-use agreement, and compliance with applicable ethical and privacy restrictions. Raw gait videos are not publicly available and cannot be shared because they contain potentially identifiable human-movement information and mental-health-related data, posing privacy and re-identification risks.
